# Production of Castor Oil in Illinois

**Published:** 1840

**Authors:** 


### PRODUCTION OF CASTOR OIL IN ILLINOIS.

Having lately had occasion to spend an hour at the village of Chester, one hundred and twenty miles above the mouth of the Ohio river, on the Illinois bank of the Mississippi, we learned from Dr. Ferris, the following facts, concerning the manufacture of castor oil in the neighborhood of that place.

About seven years ago, Richard B. Servant, Esq., introduced into Randolph county, near the town of Chester, the cultivation of the castor oil plant, *Ricinus communis*, and it has since spread over that and the adjoining counties—Washington, Franklin and Jackson. It is cultivated on their upland soils, and ripens before the frosts of autumn set in. From twenty-five to thirty bushels per acre, are obtained. It is planted and tilled in the same manner as Indian corn. A bushel of the beans yield from six to eight quarts of oil, which is obtained by cold pressure. A mill for its manufacture, was lately burned down in Chester (and will be rebuilt,) but, three others are in operation in Randolph county. Dr. Ferris exhibited to us a specimen of the oil, eighteen months old, which was limpid and of the characteristic odor. The Doctor assured us, that it was as active and unirritating as any he has ever prescribed. The quantity exported from Chester, is so great, as to surprise us. The crop of 1338, was such, that one thousand barrels were sent off in 1839; while the produce of that year, was sufficient to give for 1840, at least twelve hundred; worth to the manufacturer from a dollar to a dollar and a quarter a gallon. It is sent not only to all the principal towns of the west and south-west, but to the eastern cities. When we recollect, not many years back, the importation of *rancid* oil, in bottles, from beyond the mountains at an enormous expense, we cannot but regard the citizens of Illinois, who have effectuated this enterprize, as public benefactors, while they have made it a source of wealth to themselves. D.

